# Molecular characterization of feline immune checkpoint molecules and establishment of PD-L1 immunohistochemistry for feline tumors

**DOI:** 10.1371/journal.pone.0281143

**Published:** 2023-01-26

**Authors:** Naoya Maekawa, Satoru Konnai, Yumie Asano, Takumi Otsuka, Eri Aoki, Hiroto Takeuchi, Yukinari Kato, Mika K. Kaneko, Shinji Yamada, Yumiko Kagawa, Maki Nishimura, Satoshi Takagi, Tatsuya Deguchi, Hiroshi Ohta, Takayuki Nakagawa, Yasuhiko Suzuki, Tomohiro Okagawa, Shiro Murata, Kazuhiko Ohashi

**Affiliations:** 1 Department of Advanced Pharmaceutics, Faculty of Veterinary Medicine, Hokkaido University, Sapporo, Japan; 2 Department of Disease Control, Faculty of Veterinary Medicine, Hokkaido University, Sapporo, Japan; 3 Institute for Vaccine Research and Development (HU-IVReD), Hokkaido University, Sapporo, Japan; 4 Department of Antibody Drug Development, Tohoku University Graduate School of Medicine, Sendai, Japan; 5 Department of Molecular Pharmacology, Tohoku University Graduate School of Medicine, Sendai, Japan; 6 North Lab, Sapporo, Japan; 7 Veterinary Teaching Hospital, Faculty of Veterinary Medicine, Hokkaido University, Sapporo, Japan; 8 Department of Veterinary Surgery 1, School of Veterinary Medicine, Azabu University, Sagamihara, Japan; 9 Companion Animal Internal Medicine, Department of Companion Animal Clinical Sciences, School of Veterinary Medicine, Rakuno Gakuen University, Ebetsu, Japan; 10 Laboratory of Veterinary Surgery, Graduate School of Agricultural and Life Sciences, The University of Tokyo, Bunkyo, Japan; 11 International Institute for Zoonosis Control, Hokkaido University, Sapporo, Japan; 12 Global Station for Zoonosis Control, Global Institution for Collaborative Research and Education (GI-CoRE), Hokkaido University, Sapporo, Japan; 13 International Affairs Office, Faculty of Veterinary Medicine, Hokkaido University, Sapporo, Japan; King Faisal Specialist Hospital and Research Center, SAUDI ARABIA

## Abstract

Spontaneous tumors are a major cause of death in cats. Treatment of human tumors has progressed dramatically in the past decade, partly due to the success of immunotherapies using immune checkpoint inhibitors, such as anti-programmed death 1 (PD-1) and anti-PD-ligand 1 (PD-L1) antibodies. However, little is known about the PD-1 pathway and its association with tumor disease in cats. This study investigated the applicability of anti-PD-1/PD-L1 therapy in feline tumors. We first determined the complete coding sequence of feline *PD-L1* and *PD-L2*, and found that the deduced amino acid sequences of feline PD-L1/PD-L2 share high sequence identities (66–83%) with orthologs in other mammalian species. We prepared recombinant feline PD-1, PD-L1, and PD-L2 proteins and confirmed receptor–ligand binding between PD-1 and PD-L1/PD-L2 using flow cytometry. Next, we established an anti-feline PD-L1 monoclonal antibody (clone CL1Mab-7) to analyze the expression of PD-L1. Flow cytometry using CL1Mab-7 revealed the cell surface expression of PD-L1 in a feline macrophage (Fcwf-4) and five mammary adenocarcinoma cell lines (FKNp, FMCm, FYMp, FONp, and FONm), and showed that PD-L1 expression was upregulated by interferon-γ stimulation. Finally, immunohistochemistry using CL1Mab-7 also showed PD-L1 expression in feline squamous cell carcinoma (5/5, 100%), mammary adenocarcinoma (4/5, 80%), fibrosarcoma (5/5, 100%), and renal cell carcinoma (2/2, 100%) tissues. Our results strongly encourage further investigations of the PD-1/PD-L1 pathway as a potential therapeutic target for feline tumors.

## Introduction

Tumor treatment in humans has progressed dramatically in the past decade, partly due to the success of molecular-targeted therapies and immunotherapies. Among the emerging therapies, immune checkpoint inhibitors (ICIs), such as anti-programmed death 1 (PD-1) and anti-PD-ligand 1 (PD-L1) antibodies, have shown great promise in the treatment of various malignancies including melanoma, non-small-cell lung cancer (NSCLC), and renal-cell cancer [1, 2]. Cats develop spontaneous tumors typically at >8 years of age, and malignant tumors are diagnosed 4.6-fold more often than benign tumors [3]. Feline tumors are treated with surgery, radiation, and chemotherapy; however, despite advances in veterinary care, neoplasia-related deaths account for 10.8% of the mortality in cats, ranking fourth following trauma (12.2%), renal disorder (12.1%), and non-specific illness (11.2%) [4]. Therefore, the establishment of immunotherapies for feline tumors is urgently needed as an additional/alternative option to conventional therapies.

The PD-1/PD-L1 pathway is an important suppressive mechanism for T cell-mediated immunity. PD-1 is an immunoinhibitory receptor expressed on T cells that attenuates antigen-specific responses, such as cell proliferation, cytokine secretion, and cytotoxicity [5, 6]. The expression of PD-L1, a PD-1 ligand, can be found in both hematopoietic and non-hematopoietic cell types, whereas the expression of PD-L2, the other ligand, is restricted to immune cells, such as dendritic cells and macrophages [5]. PD-L1 overexpression is often reported in various human tumors and its expression is correlated with poor prognosis in cancer patients [7–11]. The blockade of PD-1/PD-L1 using antibodies induces robust antitumor immune responses by reinvigorating T cell effector functions [6, 12], which has promoted the standard of care for various malignancies in humans. In cats, limited information is available on the PD-1/PD-L1 pathway and its association with tumor disease is unclear. Folkl *et al*. [13] reported the complete feline *PD-1* and partial *PD-L1* mRNA coding sequences (CDS) and found upregulation of PD-1/PD-L1 proteins in the lymphocytes of cats chronically infected with feline immunodeficiency virus. The complete CDS for feline *PD-L2* mRNA has been registered in the NCBI GenBank database by the same authors (accession number: NM_001290244.1); however, the experimental procedures have not yet been reported, and characterization of its protein is yet to be performed. Recently, an immunohistochemical study using anti-human PD-L1 monoclonal antibody revealed that HER2-positive and triple-negative normal-like feline mammary carcinomas express PD-L1 [14], while PD-L1 expression in other feline tumors remains unknown. Thus, further genetic and functional characterization of feline PD-L1/PD-L2, as well as the analysis of PD-L1 expression in various feline malignant tumors, is required to develop effective feline ICI therapies.

PD-L1 is a useful biomarker of the clinical response of tumors to anti-PD-1/PD-L1 antibody therapies in humans. In a clinical study of melanoma, NSCLC, colorectal cancer, renal-cell cancer, or castration-resistant prostate cancer, pretreatment tumor cell-surface expression of PD-L1 (as determined with immunohistochemistry [IHC]) was correlated with the response to PD-1 blockade, wherein 36% of patients with PD-L1-positive tumors experienced an objective response, while no (0%) patients with PD-L1-negative tumors responded to the treatment [1]. Another study in NSCLC patients treated with anti-PD-1 antibody revealed that patients with a proportion score (percentages of neoplastic cells with membranous PD-L1 staining) of at least 50% had a higher response rate and longer progression-free/overall survival than those with a proportion score of less than 50% [15]. Accordingly, the successful application of anti-PD-1/PD-L1 antibody drugs requires immunohistochemical assessment of PD-L1 for the selection of eligible patients. Although PD-L1 IHC has some limitations as a predictive biomarker [8] and other biomarkers, such as tumor mutational burden and microsatellite instability, have been proposed [16], the establishment of a sensitive and robust PD-L1 IHC for feline tumors is of particular importance for informed selection of cat subjects that are most likely to benefit from anti-PD-1/PD-L1 therapies.

To explore the feasibility of anti-PD-1/PD-L1 therapy for feline tumors, we first determined the complete CDS of feline *PD-L1* and *PD-L2* mRNA and compared the deduced amino acid sequences with known orthologs in other mammalian species. Recombinant proteins of feline PD-1, PD-L1, and PD-L2 were prepared for molecular characterization, and receptor–ligand binding was assessed in an expressing cell-based assay. Then, an anti-feline PD-L1 monoclonal antibody (CL1Mab-7) was established and its applicability for expression analysis was evaluated by flow cytometry using feline macrophage and mammary adenocarcinoma cell lines. Finally, IHC using CL1Mab-7 was developed to probe PD-L1 expression in various feline malignant tumors.

## Materials and methods

### Feline samples

The use of animal-derived samples throughout the study was approved by the Institutional Animal Care and Use Committee, Faculty of Veterinary Medicine, Hokkaido University (approval #20–0093), which has been fully accredited by the Association for Assessment and Accreditation of Laboratory Animal Care International. No animal was sacrificed for this study. Testis tissue samples were freshly resected from a mixed-breed cat under general anesthesia at the Veterinary Teaching Hospital, Faculty of Veterinary Medicine, Hokkaido University. Peripheral blood samples were obtained from healthy cats (mixed breed) maintained at the Experimental Animal Facility, Faculty of Veterinary Medicine, Hokkaido University or a veterinary hospital in Sapporo, Japan. Formalin-fixed and paraffin-embedded (FFPE) feline tissues were obtained from a commercial pathology laboratory (North Lab, Hokkaido, Japan).

### Cell culture

Peripheral blood mononuclear cells (PBMCs) were obtained from heparinized blood samples by density gradient centrifugation on Percoll (GE Healthcare, Buckinghamshire, UK) and cultured in RPMI 1640 medium (Sigma-Aldrich, St. Louis, MO, USA) supplemented with 10% fetal bovine serum (FBS; Thermo Fisher Scientific, Waltham, MA, USA), 2 mM L-glutamine, 200 μg/mL streptomycin, 200 U/mL penicillin (Thermo Fisher Scientific), and 5 μg/mL concanavalin A (ConA; Sigma-Aldrich) for 10 h at 37°C and 5% CO_2_. ExpiCHO-S cells (Thermo Fisher Scientific) were cultured on an orbital shaker (125 rpm) at 37°C and 8% CO_2_ in ExpiCHO Expression Medium (Thermo Fisher Scientific). The feline macrophage cell line Fcwf-4 [Fcwf] (ATCC CRL-2787) was cultured in Eagle’s Minimum Essential Medium (ATCC, Manassas, VA, USA) containing 10% FBS (Thermo Fisher Scientific) at 37°C and 5% CO_2_. The feline mammary adenocarcinoma cell lines FKNp, FMCp, FMCm, FYMp, FONp, and FONm [17] were cultured in RPMI 1640 medium (Sigma-Aldrich) supplemented with 10% FBS (Thermo Fisher Scientific), 2 mM L-glutamine, 200 μg/mL streptomycin, and 200 U/mL penicillin (Thermo Fisher Scientific) at 37°C and 5% CO_2_. Cell lines were cultured with 100 ng/mL recombinant feline interferon-gamma (IFN-γ; Kingfisher Biotech, St. Paul, MN, USA) for 24 h before analysis, where indicated.

### Nucleotide sequencing, alignment, and phylogenetic analysis

To determine the complete CDS of feline *PD-L1*, we designed primers for rapid amplification of cDNA ends (RACE; fePD-L1_5′GSP1–3, fePD-L1_3′GSP1, and -2) based on a previously reported partial sequence of feline *PD-L1* (GenBank accession number: EU246348.2). Total RNA was extracted from cat testes using TRI reagent (Molecular Research Center, Cincinnati, OH, USA), and 3′ and 5′ unknown sequences were amplified using 3′ and 5′ RACE System for Rapid Amplification of cDNA Ends (Thermo Fisher Scientific) and TaKaRa Ex Taq polymerase (Takara Bio, Shiga, Japan). The amplicons were purified, cloned into pGEM-T Easy Vector (Promega, Madison, WI, USA), and sequenced using GenomeLab GeXP Genetic Analysis System (SCIEX, Framingham, MA, USA). Based on the obtained sequence, a primer pair was designed to amplify the whole CDS of feline *PD-L1* (fePD-L1_F and R), and polymerase chain reaction (PCR) was performed using TaKaRa Ex Taq polymerase (Takara Bio) and cDNA templates synthesized from ConA-stimulated feline PBMCs, as previously described [18]. Nucleotide sequences of the amplicons were determined as described above. Similarly, to determine the complete CDS of feline *PD-L2*, a primer pair was designed based on NM_001290244.1 (fePD-L2_F and R), PCR was performed using cDNAs of ConA-stimulated feline PBMCs, and the nucleotide sequences of the amplicons were determined as described above. The CDSs of feline *PD-L1* and *PD-L2* were translated into deduced amino acid sequences and aligned with known orthologs in other mammalian species using BioEdit [19]. The signal peptide, transmembrane domain, and other conserved domains were predicted using SignalP 6.0 (https://services.healthtech.dtu.dk/service.php?SignalP), TMHMM 2.0 (https://services.healthtech.dtu.dk/service.php?TMHMM-2.0), and CD-Search (https://www.ncbi.nlm.nih.gov/Structure/cdd/wrpsb.cgi), respectively. Potential N-linked glycosylation sites were predicted using NetNGlyc-1.0 (https://services.healthtech.dtu.dk/service.php?NetNGlyc-1.0). The percentages of identical and positive matches between amino acid sequences were calculated using Protein BLAST (blastp; https://blast.ncbi.nlm.nih.gov/Blast.cgi?PAGE=Proteins). Unrooted neighbor-joining phylogenetic trees were inferred using MEGA version 6.06 [20, 21]. Nucleotide sequences of feline PD-L1 and PD-L2 were submitted to the DDBJ/EMBL-Bank/GenBank database under accession numbers LC735019, LC735020, and LC735021. Primer sequences used in this study are listed in Table 1.

**Table 1:** PCR primer sequences.

Primer name	Nucleotide sequence (5′–3′)	Remark
fePD-L1_5′GSP1	CTAGAATCATGAAGTGA	EU246348.2
fePD-L1_5′GSP2	TTGATTCTCAGCGTGCTGGT	EU246348.2
fePD-L1_5′GSP3	GCCATAGCCAATCAAGCAGC	EU246348.2
fePD-L1_3′GSP1	GCTGCTTGATTGGCTATGGC	EU246348.2
fePD-L1_3′GSP2	TGCTGAGTTGGTCATCCCAG	EU246348.2
fePD-L1_F	CTCCCCGCCGGCAGAAAA	
fePD-L1_R	CTGGTCATGCTTACCCCTGACG	
fePD-L2_F	TCAGATCGGGTACAGAGC	NM_001290244.1
fePD-L2_R	TTCAGAGTCCAGAACATTCTC	NM_001290244.1
fePD-1–EGFP_F	CGGAATTCATGGGGACCCCACGGGCGCC	NM_001145510.1, EcoRI
fePD-1–EGFP_R	CGGGATCCCGAGGGGCCAAGGGCAGGGTC	NM_001145510.1, BamHI
fePD-L1–EGFP_F	GAAGATCTATGAGGATATTTAGTGTCTT	BglII
fePD-L1–EGFP_R	CGGAATTCCGTCTCCTCAAATTGTAGAT	EcoRI
fePD-1–Ig_F	CGCGGCTAGCATGGGGACCCCACGGGCGC	NM_001145510.1, NheI
fePD-1–Ig_R	CGCGGATATCCAGCCCCTGGCCTTGGCCG	NM_001145510.1, EcoRV
fePD-L1–Ig_F	CGCGGCTAGCATGAGGATATTTAGTGTCT	NheI
fePD-L1–Ig_R	CGCGGATATCCCTCTCATTTGCTGGAACC	EcoRV
fePD-L2–Ig_F	CGCGGATATCATGTTCCTCCTGCTGATGTT	EcoRV
fePD-L2–Ig_R	CGGGGTACCATTTGAAGGGATCTTGGGTT	KpnI

GenBank accession numbers for the reference sequences are shown. Restriction enzyme recognition sites are underlined.

### Transient expression of recombinant proteins

To prepare feline PD-1 or PD-L1-expressing cells, we constructed expression vectors for feline PD-1 or PD-L1 fused to the C-terminal enhanced green fluorescent protein (fePD-1–EGFP; fePD-L1–EGFP) using pEGFP-N2 vector (Clontech, Palo Alto, CA, USA). Nucleotide sequences encoding feline PD-1 (NM_001145510.1) and PD-L1 were amplified by PCR using specific primers with restriction enzyme cleavage sites (fePD-1–EGFP_F and R; fePD-L1–EGFP_F and R, Table 1), and subcloned into the multicloning site of the pEGFP-N2 vector. The expression plasmids were purified using FastGene Xpress Plasmid PLUS Kit (Nippon Genetics, Tokyo, Japan) and stored at −20°C until use. ExpiCHO-S cells were transfected with the plasmid using ExpiFectamine CHO Transfection Kit (Thermo Fisher Scientific) and cultured for 24 h prior to analysis. The subcellular localization of fePD-1–EGFP and fePD-L1–EGFP was visualized using ZOE Fluorescent Cell Imager (Bio-Rad, Hercules, CA, USA).

To prepare soluble feline PD-1, PD-L1, and PD-L2 proteins, we constructed expression vectors for feline PD-1, PD-L1, or PD-L2 extracellular region fused to the C-terminal rabbit IgG Fc region (fePD-1–Ig, fePD-L1–Ig, and fePD-L2–Ig) using pCXN2.1 (+) vector (kindly provided by Dr. T. Yokomizo, Juntendo University, Tokyo, Japan) [22]. Nucleotide sequences encoding the extracellular regions of feline PD-1 (1–169), PD-L1 (1–237), and PD-L2 (1–219) were amplified by PCR using specific primers with restriction enzyme cleavage sites (fePD-1–Ig_F and R, fePD-L1–Ig_F and R, and fePD-L2–Ig_F and R; Table 1), and subcloned into the multicloning site of the pCXN2.1 (+) vector containing a gene cassette encoding the Fc region of rabbit IgG. The expression plasmids were purified using FastGene Xpress Plasmid PLUS Kit (Nippon Genetics) or NucleoBond Xtra Midi (Takara Bio) and stored at −20°C until use. ExpiCHO-S cells were transfected with the plasmid using ExpiFectamine CHO Transfection Kit (Thermo Fisher Scientific) and cultured for 10 d. Recombinant fusion proteins were purified from the culture supernatant by protein A affinity chromatography using Ab-Capcher ExTra (ProteNova, Kagawa, Japan). After elution with Pierce IgG Elution Buffer (Thermo Fisher Scientific), the buffer was replaced with phosphate-buffered saline (PBS; FUJIFILM Wako Pure Chemical, Osaka, Japan) using PD MidiTrap G25 (Cytiva, Tokyo, Japan). The protein concentration was measured using Pierce BCA Protein Assay Kit (Thermo Fisher Scientific). Sodium dodecyl sulfate polyacrylamide gel electrophoresis (SDS-PAGE) was performed using 2× Laemmli sample buffer (Bio-Rad) prepared with 2-mercaptoethanol (reducing conditions; FUJIFILM Wako Pure Chemical) or distilled water (non-reducing conditions). Recombinant fusion proteins were incubated at 96°C for 5 min in sample buffer and separated by electrophoresis using SuperSep Ace 5–20% gradient gel (FUJIFILM Wako Pure Chemical). Precision Plus Protein Dual Color Standards (Bio-Rad) were used as molecular weight markers. Protein bands were visualized by Coomassie brilliant blue (CBB) staining using Quick-CBB kit (FUJIFILM Wako Pure Chemical).

### Monoclonal antibody against feline PD-L1

To establish the anti-feline PD-L1 monoclonal antibody CL1Mab-7, BALB/c mice (4-week-old, female; CLEA Japan, Tokyo, Japan) were immunized intraperitoneally with 100 μg fePD-L1–Ig formulated with Imject Alum Adjuvant (Thermo Fisher Scientific). After the second booster administration, splenocytes were harvested and fused with P3X63Ag8U.1 (ATCC CRL-1597) cells using PEG1500 (Roche Diagnostics, Indianapolis, IN, USA) to generate a hybridoma pool. Cell cloning was performed by limiting dilution and monoclonal antibodies were purified from the culture supernatant using Protein G Sepharose 4 Fast Flow (GE Healthcare). Isotype-specific secondary antibodies (Southern Biotech, Birmingham, AL, USA) were used to identify the CL1Mab-7 antibody (sub)class (mouse IgG_1_, κ).

### Flow cytometry

To examine whether feline PD-1 binds to PD-L1/PD-L2, 2 × 10^5^ fePD-1–EGFP- and fePD-L1–EGFP-expressing cells were incubated for 30 min with 10 μg/mL of either fePD-1–Ig, fePD-L1–Ig, or fePD-L2–Ig at room temperature (RT), followed by another 30 min incubation with Alexa Fluor 647-conjugated F(ab′)_2_-goat anti-rabbit IgG (H+L) secondary antibody (Thermo Fisher Scientific). Rabbit IgG (Southern Biotech) was used as a control protein. Cell fluorescence was analyzed using BD FACSLyric system (BD Biosciences, Franklin Lakes, NJ, USA).

To examine PD-L1 expression on cell lines, 2 × 10^5^ cells were incubated for 30 min with 10 μg/mL CL1Mab-7 or mouse IgG_1_κ isotype-matched control antibody (15H6; Southern Biotech) at RT, followed by another 30 min incubation with Alexa Fluor 647-conjugated F(ab′)_2_-goat anti-mouse IgG (H+L) secondary antibody (Thermo Fisher Scientific). Cell fluorescence was analyzed using BD FACSLyric system (BD Biosciences). For fePD-1–EGFP- and fePD-L1–EGFP-expressing cells, only EGFP-positive cells were gated and subjected to further analysis.

### Immunohistochemistry

We prepared 4-μm-thick sections of FFPE feline squamous cell carcinoma (*n* = 5), mammary adenocarcinoma (*n* = 5), fibrosarcoma (*n* = 5), and renal cell carcinoma (*n* = 3) tissue samples, and antigen retrieval was performed twice by microwave treatment for 5 min in Tris-EDTA buffer (pH 9.0; Aligent Technologies, Santa Clara, CA, USA). Endogenous peroxidase activity was blocked by incubating sections in methanol containing 0.3% hydrogen peroxide. Sections were incubated with 10 μg/mL CL1Mab-7 or mouse IgG_1_κ isotype-matched control antibody (MG1-45; BioLegend, San Diego, CA, USA) at RT for 30 min, followed by another 30 min incubation with Histofine simple stain MAX PO (MULTI) (Nichirei, Tokyo, Japan). A chromogenic reaction was developed using 3, 3′-diaminobenzidine tetrahydrochloride (Nichirei). Mayer’s hematoxylin was used as a counterstain.

## Results

### Characterization of feline *PD-L1* and *PD-L2*

**Fig 1:**
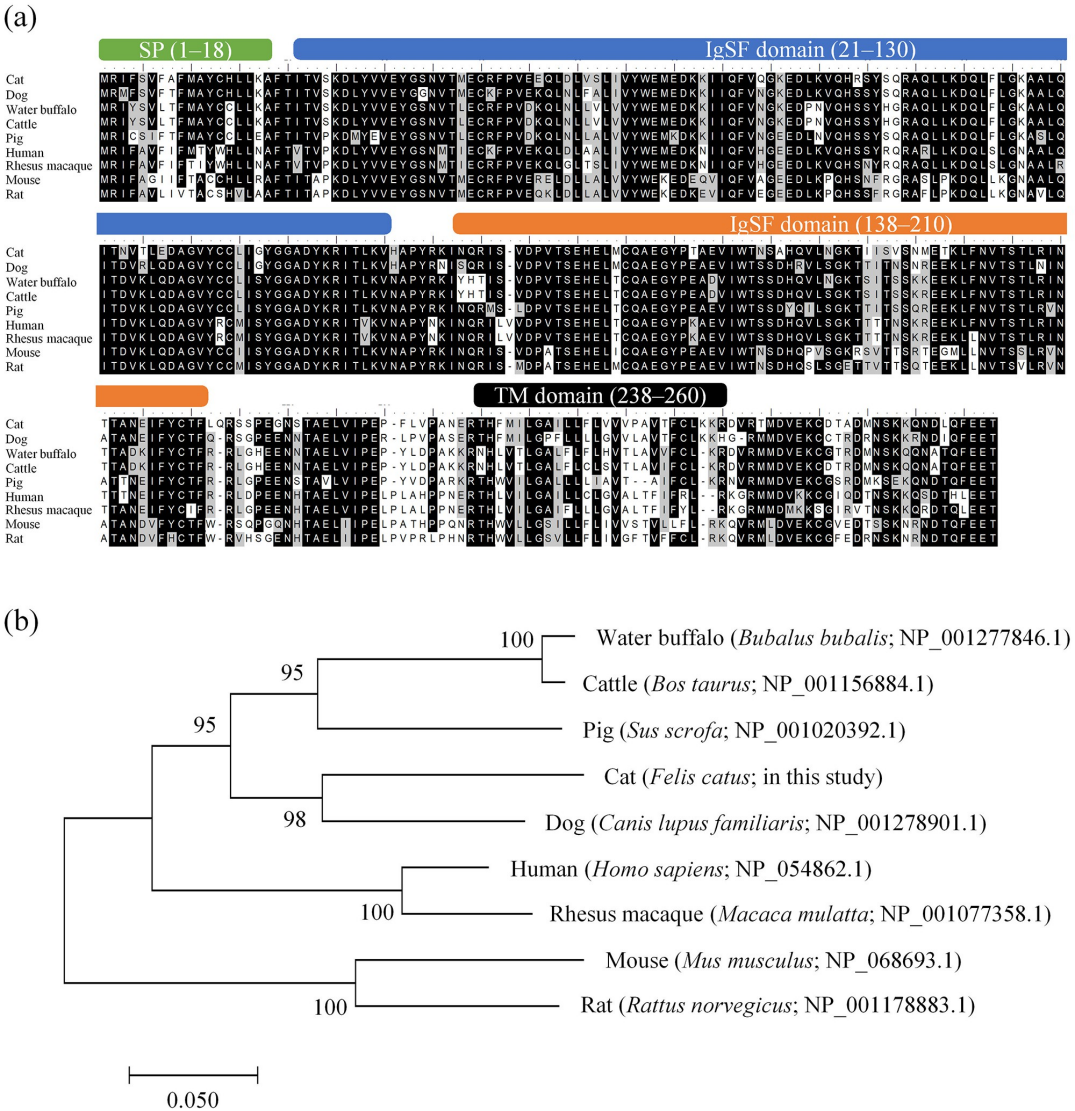
Identification of feline *PD-L1* mRNA sequence. (a) Multiple sequence alignment of PD-L1 amino acid sequences in various mammalian species. The predicted signal peptide (SP), immunoglobulin superfamily (IgSF) domains, and transmembrane (TM) domain are indicated with numbers representing the positions of amino acid residues. (b) Phylogenetic tree based on the feline PD-L1 amino acid sequence in relation to those of other mammalian species. The tree was inferred using the neighbor-joining method with a bootstrap of 1,000 replicates. Numbers next to the branches indicate the bootstrap percentage. The scale bar indicates the divergence time.

**Fig 2:**
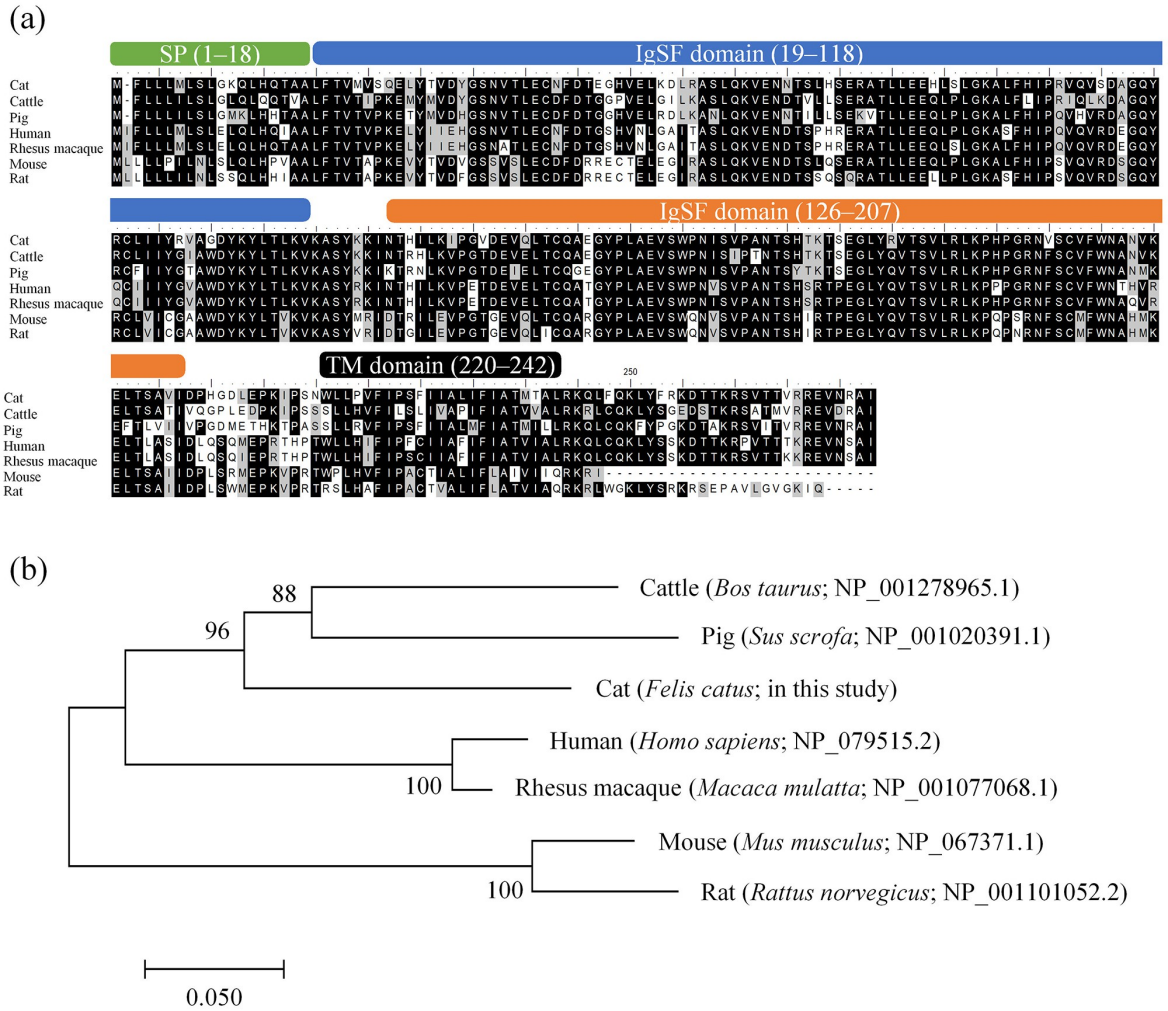
Identification of feline *PD-L2* mRNA sequence. (a) Multiple sequence alignment of PD-L2 amino acid sequences in various mammalian species. The SP, IgSF domains, and TM domain are indicated with numbers representing the positions of amino acid residues. (b) Phylogenetic tree (inferred as described in Fig 1) of the feline PD-L2 amino acid sequence in relation to those of other mammalian species. Numbers next to the branches indicate the bootstrap percentage. The scale bar indicates the divergence time.

To date, the complete nucleotide sequences of feline *PD-L1* and *PD-L2* mRNAs have not been reported in the literature, whereas only a partial sequence of *PD-L1* and a complete sequence of *PD-1* have been described [13]. We first determined the mRNA sequences of feline *PD-L1* and *PD-L2* using cDNAs synthesized from PBMCs of mixed-breed cats. The complete CDS of feline *PD-L1* and *PD-L2* was 876 and 819 bp in length, encoding 291 and 272 polypeptide sequences, respectively. The *PD-L1* mRNA sequence was identical among four individual cats, whereas the *PD-L2* mRNA sequence contained a single nucleotide polymorphism between the two cats tested (C520A; silent mutation). The deduced amino acid sequence of feline PD-L1 and PD-L2 showed high sequence identity with orthologs in other mammalian species (66–83%, Table 2). Both feline PD-L1 and PD-L2 were predicted to contain a signal peptide, two immunoglobulin superfamily (IgSF) domains, and a transmembrane domain (Figs 1a and 2a). In the phylogenetic analyses, feline PD-L1 and PD-L2 formed a cluster with orthologs in Perissodactyla (e.g., cattle and pig) and Carnivora (dog), and were relatively distant in relation to Primates (human and rhesus macaque) and Rodentia (mouse and rat; Figs 1b and 2b).

**Table 2:** Amino acid sequence similarities of feline PD-L1/PD-L2 with other mammals.

	PD-L1 identities (%)	PD-L1 positives (%)	PD-L2 identities (%)	PD-L2 positives (%)
Dog	83	88	NA	NA
Water buffalo	77	84	NA	NA
Cattle	77	84	78	85
Pig	74	86	77	84
Human	73	80	73	81
Rhesus macaque	71	78	76	83
Mouse	67	79	69	80
Rat	67	79	66	79

Percentages of identical and positive (allowing conservative substitution) matches in the amino acid sequence of feline PD-L1 and PD-L2 are calculated in relation to orthologs from other mammalian species by Protein BLAST (blastp). NA: not available.

### Characterization of feline PD-1, PD-L1, and PD-L2

**Fig 3:**
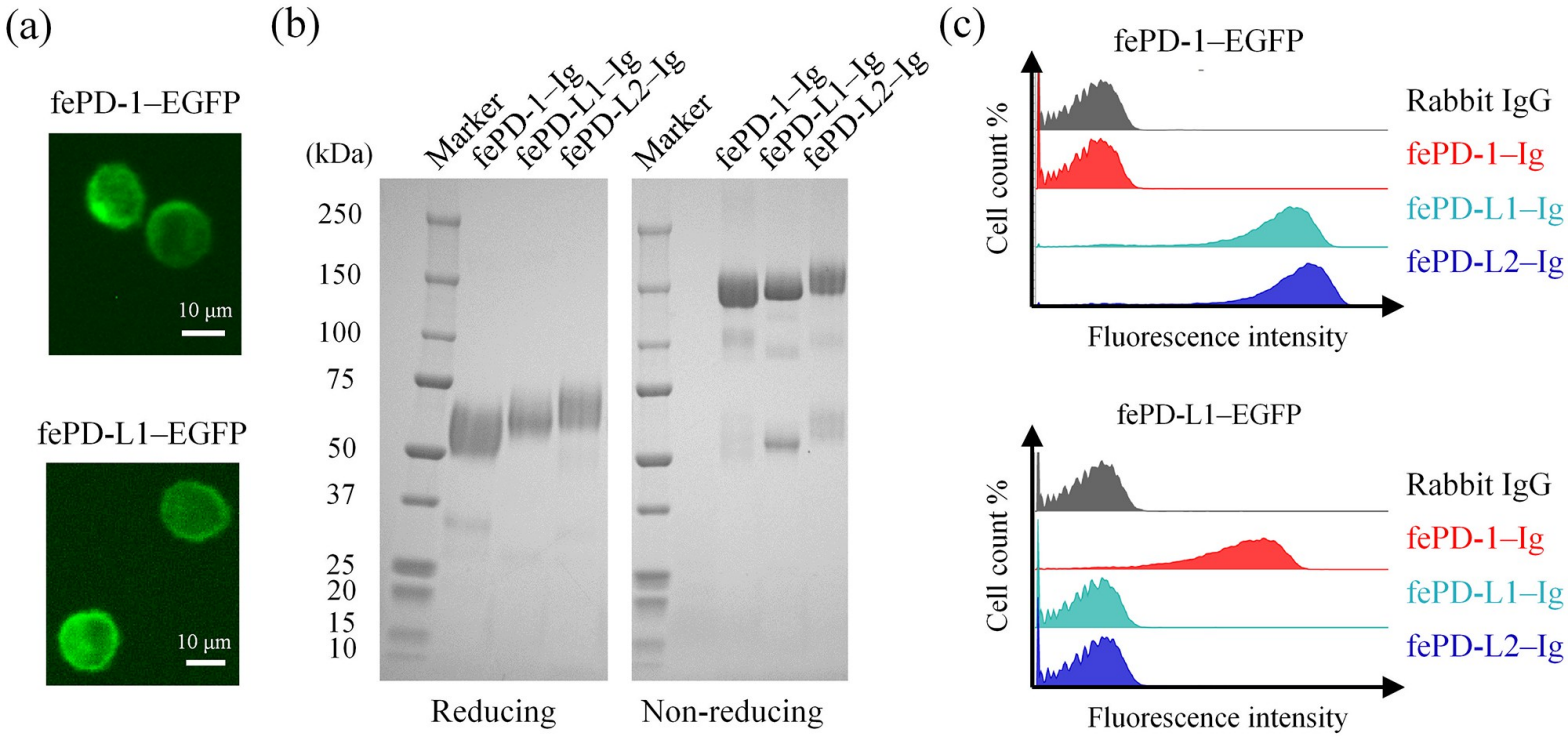
Characterization of feline PD-1/PD-L1/PD-L2 proteins. (a) Preparation of fePD-1–EGFP- and fePD-L1–EGFP-expressing cells. Cells were harvested and observed under a fluorescent microscope at 24 h post transfection. (b) Preparation of fePD-1–Ig, fePD-L1–Ig, and fePD-L2–Ig. The purified Fc-fusion proteins were separated under reducing (left) or non-reducing (right) conditions by SDS-PAGE. The proteins were visualized by CBB staining. (c) Binding of Fc-fusion proteins to fePD-1–EGFP- and fePD-L1–EGFP-expressing cells. Cells were incubated with 10 μg/mL of each Fc-fusion protein and their binding was detected using anti-rabbit IgG secondary antibody. Rabbit IgG was used as a negative control.

Next, we expressed recombinant feline PD-1 and PD-L1 as C-terminal EGFP-fusion proteins and examined their subcellular localization using fluorescence microscopy. In both fePD-1–EGFP- and fePD-L1–EGFP-expressing cells, EGFP fluorescence was concentrated at the cell membrane, indicating cell surface expression of the recombinant fusion proteins (Fig 3a). To examine the binding between PD-1 and PD-L1/PD-L2, the extracellular regions of PD-1, PD-L1, and PD-L2 were expressed as soluble rabbit IgG Fc fusion proteins. Purified fePD-1–Ig, fePD-L1–Ig, and fePD-L2–Ig migrated at approximately 60 kDa and 140 kDa under reducing and non-reducing conditions, respectively, suggesting dimer formation by a disulfide bond at the hinge region of rabbit IgG Fc under non-reducing conditions (Fig 3b). The theoretical molecular weights (as monomers) of fePD-1–Ig, fePD-L1–Ig, and fePD-L2–Ig, calculated from the deduced amino acid sequences, were 42.3, 50.9, and 48.3 kDa, respectively. Because four, five, and five potential N-glycosylation sites were found in the extracellular regions of feline PD-1, PD-L1, and PD-L2, respectively, these proteins were expected to be highly glycosylated, which influences migration in SDS-PAGE. Then, the bindings of Fc-fusion proteins to fePD-1–EGFP- and fePD-L1–EGFP-expressing cells were assessed by flow cytometry. As expected, both fePD-L1–Ig and fePD-L2–Ig were bound to fePD-1–EGFP-expressing cells, and only fePD-1–Ig was bound to fePD-L1–EGFP-expressing cells (Fig 3c), suggesting receptor–ligand interactions between feline PD-1 and PD-L1/PD-L2.

### PD-L1 expression in feline macrophage and mammary adenocarcinoma cell lines

**Fig 4:**
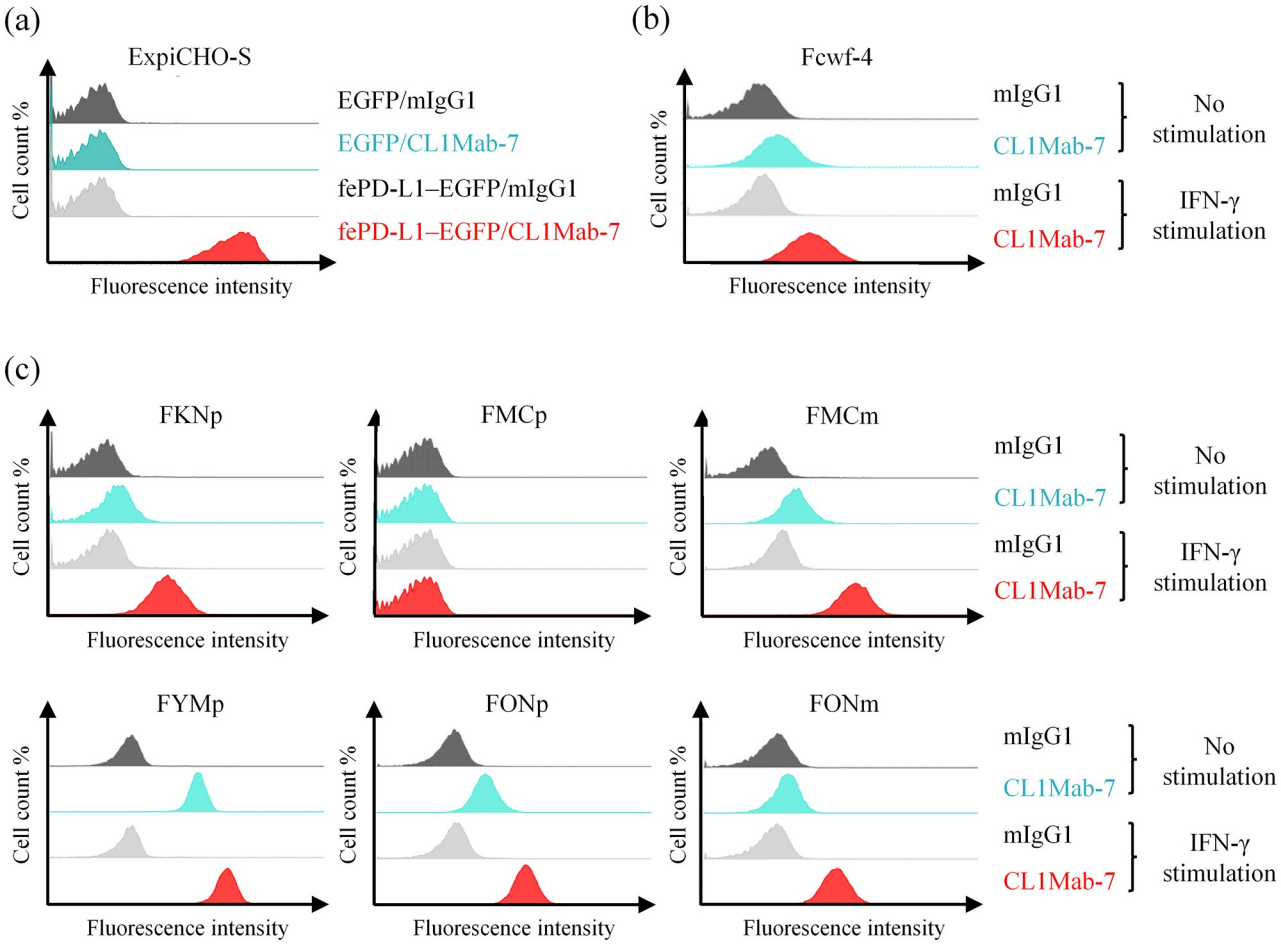
PD-L1 expression in feline macrophage and mammary adenocarcinoma cell lines. (a) Binding of the anti-feline PD-L1 monoclonal antibody CL1Mab-7 to fePD-L1–EGFP-expressing cells. Cells were transfected with EGFP (mock) or fePD-L1–EGFP expression vector and analyzed by flow cytometry at 24 h post transfection. (b) PD-L1 expression in the feline macrophage cell line Fcwf-4. (c) PD-L1 expression in feline mammary adenocarcinoma cell lines FKNp, FMCp, FMCm, FYMp, FONp, and FONm. Cells were incubated with 100 ng/mL of recombinant feline IFN-γ for 24 h where indicated. Mouse IgG_1_ (mIgG_1_) was used as an isotype-matched negative control antibody.

To investigate PD-L1 expression in feline tumors, the anti-feline PD-L1 monoclonal antibody CL1Mab-7 was established by immunizing mice with fePD-L1–Ig. The binding specificity of CL1Mab-7 was confirmed by flow cytometry, in which CL1Mab-7 reacted with fePD-L1–EGFP-expressing cells, while no binding was observed in control EGFP-expressing cells (mock, transfected with an empty vector; Fig 4a). Then, we tested whether CL1Mab-7 could detect the native PD-L1 protein expressed on the surface of feline cells. Consistent with previous reports showing that monocytes/macrophages constitutively express PD-L1 [5, 7, 23] and IFN-γ is a potent inducer of PD-L1 expression [7, 24], CL1Mab-7 showed slight binding to Fcwf-4 cells, and its binding was increased by treatment with IFN-γ (Fig 4b). We next examined PD-L1 expression on feline mammary adenocarcinoma cell lines using CL1Mab-7 via flow cytometry. Among the six cell lines, FKNp, FMCm, FYMp, FONp, and FONm expressed PD-L1 at various levels; IFN-γ stimulation enhanced PD-L1 expression on these five cell lines. The other cell line, FMCp, did not express PD-L1 even in the presence of IFN-γ (Fig 4c).

### PD-L1 expression in feline malignant tumor tissues

**Fig 5:**
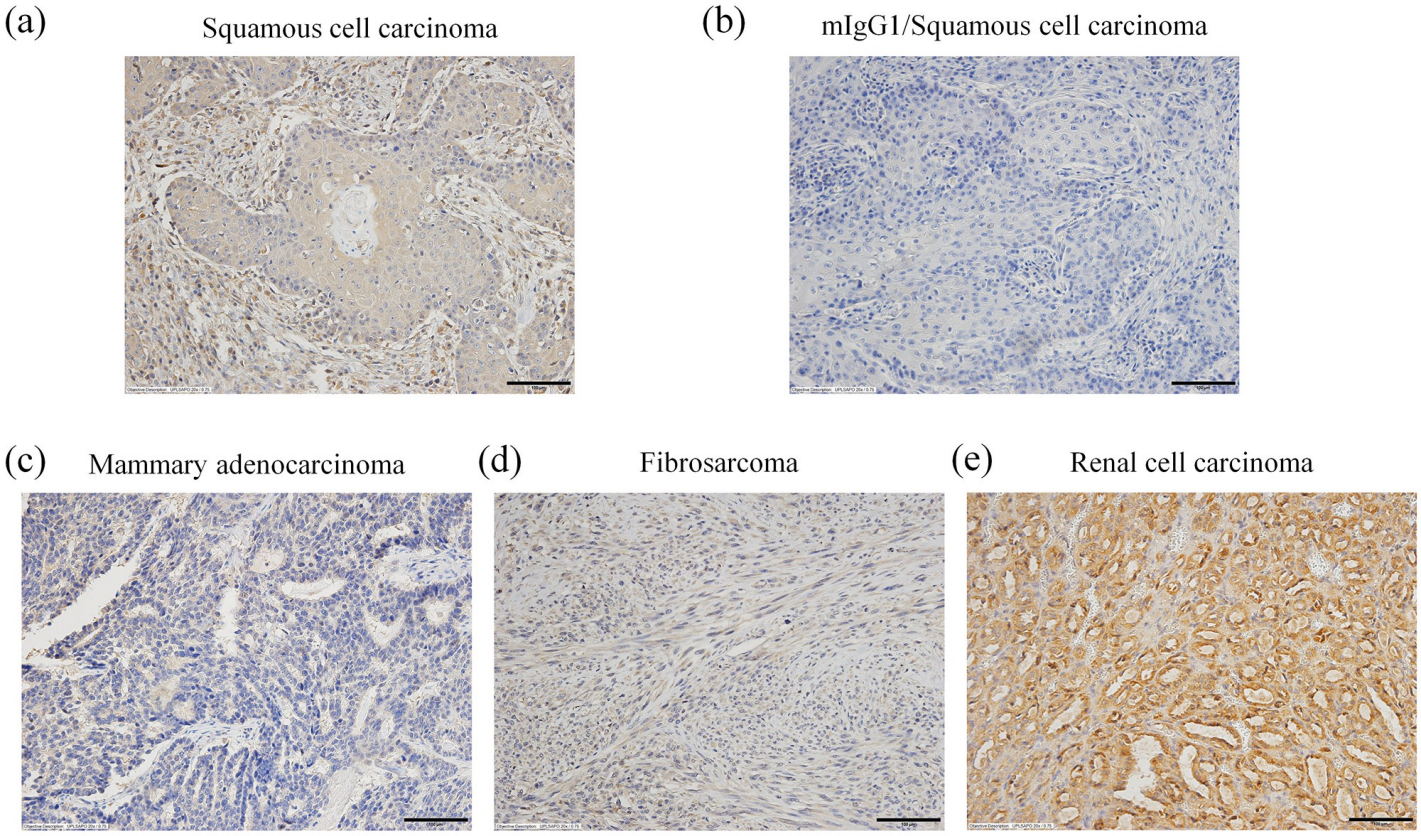
PD-L1 immunohistochemistry (IHC) for feline malignant tumors. (a) IHC staining of feline squamous cell carcinoma using anti-feline PD-L1 monoclonal antibody CL1Mab-7. (b) IHC staining of feline squamous cell carcinoma using mIgG_1_. (c) CL1Mab-7 IHC staining of feline mammary adenocarcinoma. (d) CL1Mab-7 IHC staining of feline fibrosarcoma. (e) CL1Mab-7 IHC staining of feline renal cell carcinoma. Scale bar, 100 μm.

Finally, PD-L1 IHC for FFPE feline tumor tissue was established using CL1Mab-7. In contrast to the isotype-matched control antibody (mouse IgG_1_), CL1Mab-7 produced specific positive signals in feline squamous cell carcinoma (Fig 5a and 5b). Among the five squamous cell carcinoma, five mammary adenocarcinoma, five fibrosarcoma, and three renal cell carcinoma samples tested, PD-L1 was detected in tumor cells of five (100%), four (80%), five (100%), and three (100%) samples, respectively (Fig 5c–5e, Table 3). The normal tubular epithelium was also positive for PD-L1 in renal cell carcinoma specimens. Tumor cells were stained intracellularly and showed faint membrane staining in most specimens, with stromal tissues surrounding the tumor cells being mostly negative for PD-L1.

**Table 3:** Summary of PD-L1 immunohistochemistry in feline malignant tumors.

Tumor type	No. of positive/tested cases	Positive rate
Squamous cell carcinoma	5/5	100%
Mammary adenocarcinoma	4/5	80%
Fibrosarcoma	5/5	100%
Renal cell carcinoma	3/3	100%

## Discussion

ICIs, such as anti-PD-1/PD-L1 antibodies, have become indispensable for the treatment of various malignancies in humans. Recently, ICIs have also been developed for use in veterinary medicine. In cattle infected with bovine leukemia virus, anti-PD-1/PD-L1 antibody treatment has been shown to reactivate antigen-specific T cell responses and reduce proviral load [25, 26]. The therapeutic potential of anti-PD-L1 antibody has also been suggested for bovine mycoplasmosis [27] and Johne’s disease [28], implying its broad applicability for chronic infections in cattle. Moreover, in dogs with malignant melanoma and undifferentiated sarcoma, treatment with anti-PD-L1 antibody exhibited antitumor efficacy with acceptable safety profiles [29, 30]. Because PD-L1 expression is found in several canine malignancies, including squamous cell carcinoma, mammary adenocarcinoma, transitional cell carcinoma, and hemangiosarcoma [18, 30–34], ICIs may also be effective for these tumors. Based on the similarity in tumorigenesis and the immune system among mammalian species, we hypothesized that immune checkpoint blockade could be an effective therapeutic strategy for feline tumor treatment. We found that the molecular characteristics of feline PD-1/PD-L1/PD-L2 are similar to those of its orthologs in other mammalian species in the aspects of evolutionary history, membranous expression, and receptor–ligand binding activities. The monoclonal antibody against feline PD-L1 CL1Mab-7 successfully detected the inducible expression of PD-L1 on feline cell lines, and IHC using the same antibody revealed PD-L1 expression in tissue samples of feline malignant tumors. These results strongly encourage further investigation of the PD-1/PD-L1 pathway as a therapeutic target for feline tumors.

The high sequence identities and conserved domain structures of PD-1/PD-L1/PD-L2 among mammal species suggest a functional similarity of the PD-1 pathway as a suppressive mechanism in T cell-mediated immunity. Although immunosuppressive functional motifs (immunoreceptor tyrosine-based inhibitory motif [ITIM] and immunoreceptor tyrosine-based switch motif [ITSM]) are conserved in the cytoplasmic tail of feline PD-1 [13], its signal transmission and suppression of T cell functions are yet to be investigated experimentally. Moreover, whether reactivation of T cell responses can be achieved by blocking the PD-1/PD-L1 pathway is a topic of future research. To date, no blocking antibody has been developed for feline PD-1/PD-L1, which limits the functional assessment of the PD-1 pathway in cats. We are now in progress to identify blocking antibodies from the hybridoma pool of anti-PD-L1 monoclonal antibodies.

IFN-γ treatment induced PD-L1 expression in feline macrophage and mammary adenocarcinoma cell lines, consistent with the findings in humans and dogs [7, 18, 24]. IFN-γ signaling induces interferon-responsive factor-1 (IRF-1) via the Janus kinase/signal transducer and activator of transcription (JAK/STAT) pathway, which upregulates the transcription of *PD-L1* [24, 35]. Although de novo synthesis of PD-L1 may be involved, the detailed mechanism of PD-L1 induction in feline cells remains unclear. In the human tumor microenvironment, various regulatory mechanisms of PD-L1 expression have been reported, including genomic alterations, epigenetic regulation, transcriptional regulation, post-transcriptional regulation, and post-translational modifications [36, 37]. Interestingly, one out of the six mammary adenocarcinoma cell lines (FMCp, Fig 4c) did not express PD-L1 even after IFN-γ stimulation, suggesting a defect in the expression machinery of PD-L1 or IFN-γ signaling pathway. Because a similar portion (1/5, 20%) of mammary adenocarcinoma tissues was PD-L1-negative in our IHC results, investigation of the frequency and types of, for example, genetic mutations in mammary adenocarcinoma may provide further insights into the regulatory mechanisms of PD-L1 in feline tumors.

We observed PD-L1 expression in the vast majority of feline tumor samples tested in this study. IHC using anti-PD-L1 antibody clone 22C3 revealed that 71.3%, 51.2%, and 38.1% of human squamous cell carcinomas, renal cancers, and breast cancers, respectively, were PD-L1-positive [38]. The high rates of PD-L1 expression in the current study could be an artifact of a small sample size; therefore, our results need to be verified with a larger sample size of feline tumors. Fibrosarcoma is a rare soft tissue tumor in humans that, based on limited data, expresses PD-L1 at a low positive rate (15%) [39]. Fibrosarcoma is more common in cats than in humans and is often diagnosed as a histologic subtype of feline injection site sarcomas (FISS) [40, 41]. The high frequency of PD-L1 expression in feline fibrosarcoma suggests that, in the unique etiopathogenesis of feline FISS (where chronic inflammation is thought to trigger tumorigenesis [42]), PD-L1 induction by inflammatory signaling (IFNs, IL-6, TNF-α, etc.) [37] may play a pivotal role in the escape from adaptive immune responses. Nonetheless, the detection sensitivity of PD-L1 IHC using CL1Mab-7 seemed high and sufficient, highlighting its potential for use in the screening of cats that would respond to anti-PD-1/PD-L1 therapy.

In conclusion, our molecular characterization of feline PD-1/PD-L1/PD-L2 suggests that the immunosuppressive functions of the PD-1 pathway are conserved in cats, and PD-L1 IHC revealed a potential immune evasion mechanism commonly exploited by feline tumors. Although whether feline T cell-mediated immunity can be reinvigorated by the PD-1/PD-L1 blockade remains to be elucidated, our results support further investigation of ICIs as potential immunotherapies for feline tumors.

## Supporting information

S1 Fig The original uncropped and unadjusted images for Fig 3b. (TIF)
