# Supplementary figures and images for: Molecular characterization of feline immune checkpoint molecules and establishment of PD-L1 immunohistochemistry for feline tumors
Source: PLoS One. 2023 Jan 26;18(1):e0281143. doi: 10.1371/journal.pone.0281143 (PMC9879432; doi:10.1371/journal.pone.0281143)

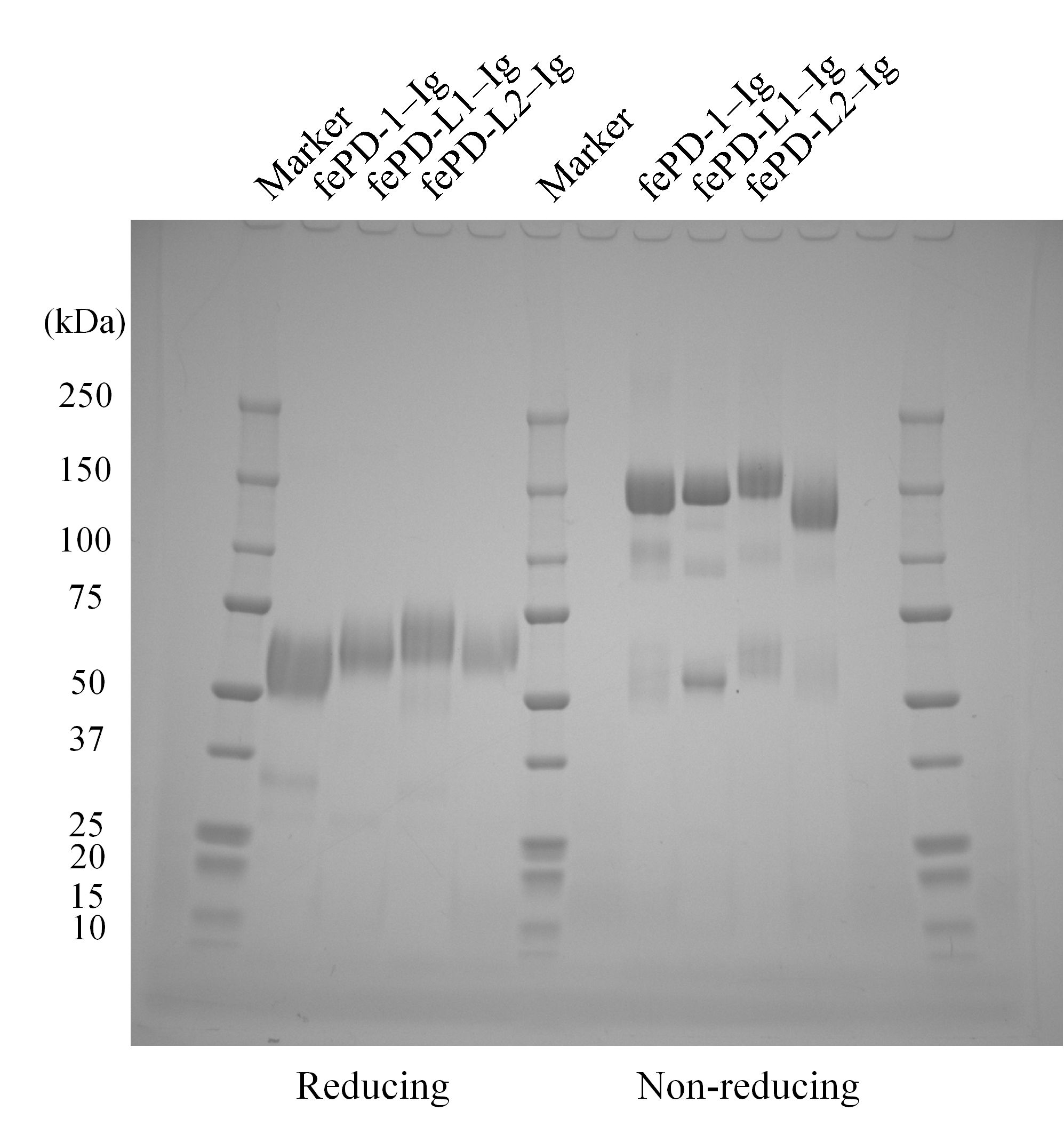

Supplement: S1 Fig — (TIF) [file pone.0281143.s001.tif]
